# What can we learn from phase II adjuvant trials in melanoma?

**DOI:** 10.1054/bjoc.2000.1277

**Published:** 2000-06-02

**Authors:** U Keilholz, S Suciu, A M M Eggermont

**Affiliations:** Immunotherapy/Chemotherapy Subgroup, EORTC Melanoma Cooperative Group, Department of Medicine III, University Hospital Benjamin Franklin, Free University Berlin, Hindenburgdamm 30, Berlin, 12200, Germany; EORTC Data Centre, av E. Mounier 83 bte 11, Brussels, 1200, Belgium; EORTC Melanoma Cooperative Group, Department of Surgical Oncology, University Hospital Rotterdam – Daniel den Hoed Cancer Center, Groene Hilledijk 301, Rotterdam, EA, 3075, The Netherlands


British Journal of Cancer (2000) 83(1), 6–7
© 2000 Cancer Research Campaign
doi: 10.1054/ bjoc.2000.1277, available online at http://www.idealibrary.com on IDEAL®

Editorial

### What can we learn from phase II adjuvant trials in melanoma?

**U Keilholz[1], S Suciu[2] and AMM Eggermont[3]**

[1]Immunotherapy/Chemotherapy Subgroup, EORTC Melanoma Cooperative Group, Department of Medicine III, University Hospital Benjamin Franklin, Free University Berlin, Hindenburgdamm 30, 12200 Berlin, Germany; [2]EORTC Data Centre, av E. Mounier, 83, bte 11, 1200 Brussels, Belgium; [3]EORTC Melanoma Cooperative Group, Department of Surgical Oncology, University Hospital Rotterdam – Daniel den Hoed Cancer Center, Groene Hilledijk 301, 3075 EA Rotterdam, The Netherlands

In this issue of the Journal, McClay and colleagues report on a clinical trial of adjuvant treatment with cisplatin and tamoxifen in 153 melanoma patients stages II to IV (McClay et al, 2000). This study has several important limitations, since it is not randomized and has no control arm. The authors nevertheless suggest a beneficial effect of treatment on disease-free and overall survival. The design of this trial deserves consideration acknowledging the information available on adjuvant treatment of melanoma at the time the study was initiated, and the report needs to be examined from the perspective of today, the time the study is reported.

### QUESTION 1

### What was the situation at the time the trial was instituted?

In 1993, there was no established standard adjuvant treatment for melanoma patients. The results of the ECOG trial 1684 on high-dose interferon were not available, and all other randomized adjuvant treatment trials with chemotherapy as well as biological agents did not suggest a benefit. The search for adjuvant treatments was clearly indicated.

For metastatic melanoma, DTIC was the only widely licensed compound. Cisplatin is another active agent, however, cisplatin-induced remissions were usually short-lived. Tamoxifen had been used in non-randomized trials as single agent or in addition to DTIC or cisplatin-based polychemotherapy. A small randomized trial reported by Cocconi suggested a survival benefit for patients with metastatic melanoma, if tamoxifen was added to DTIC (Cocconi et al, 1992). There was no further information from randomized studies concerning an effect of tamoxifen in addition to chemotherapy, although several uncontrolled phase II trials suggested a high response rate (however within the confidence limits of other polychemotherapy regimens) of treatment with chemotherapy and tamoxifen in patients with metastatic melanoma (DelPrete et al, 1984; McClay et al, 1987). Preclinical studies from the group of McClay described a synergistic interaction between cisplatin and tamoxifen (McClay et al, 1993).

In order to study a possible effect of cisplatin and tamoxifen as adjuvant treatment on survival of melanoma patients, a randomized controlled adjuvant trial protocol would have been logical in view of the information available at that time.

Knowing the information available today, there is no remaining strong rationale to study cisplatin and tamoxifen as adjuvant treatment in melanoma patients. For metastatic melanoma, a larger randomized study suggested no significant effect of cisplatin added to a combination of DTIC and vinblastin, although there was a favourable trend for an improved response-rate and time-to-progression (Jungnelius et al, 1998). If cisplatin is added to interferon and interleukin 2, the response-rate and time-to-progression can be doubled in patients with metastatic melanoma, but this is again without effect on survival, as shown in a randomized EORTC study (Keilholz et al, 1997). Regarding tamoxifen, two larger randomized studies have proven its inefficiency if added to cisplatin-containing chemotherapy in advanced melanoma (Rusthoven et al, 1996; Agarwala et al, 1999). An important recent ECOG study comparing DTIC single-agent treatment to a combination of DTIC, cisplatin, BCNU and tamoxifen revealed a (non-significant) doubling in response-rate and no effect on survival in 240 patients with metastatic melanoma (Chapman et al, 1999).

### QUESTION 2

### What type of conclusions is possible from an adjuvant non-randomized phase II clinical trial?

Uncontrolled open phase II trials are designed to test the activity and/or the toxicity of a single drug-regimen. Generally a total of 20–50 patients are recruited in these studies. Therefore this design is acceptable for treatment of patients with measurable disease. Only in case of new treatment modalities (e.g. vaccines), the proof of principle with regard to biological end-points should be assessed in the setting of phase I and II trials. For multidrug regimens, where the feasibility and activity represent the major end-points, it is recommended to perform randomized phase II/III trials. Feasibility of administration of cisplatin and tamoxifen was not an issue in this study, since, as the authors state themselves, many hundreds of patients had already received this combination, and the toxicity is well known. Treatment efficacy would therefore have been the only reasonable end-point.

McClay and colleagues do not provide a statistical rationale for their study. As a consequence, 153 patients were enrolled without justification for the patient number. Furthermore, the interval

*Correspondence to:* U Keilholz between surgery and initiation of chemotherapy is not defined. It is only stated that the scans to exclude further tumour metastases were to be done within 1 month of initiation of treatment. Unspecified is the time interval between surgery and enrolment into the study and no information is provided on the actual interval between surgery and enrolment. The possible exclusion of early relapsers might have improved survival as it may have excluded an adverse prognosis group of patients.

### QUESTION 3

### What can we learn by comparing survival data from a non-randomized adjuvant trial to historical controls?

The immediate answer of a statistician would be: nothing! There are examples in the literature, where survival analyses from uncontrolled trials in melanoma patients with metastatic disease were used to define prognostic factors for randomized studies (e.g. Balch et al, 1983; Ahmann et al, 1989; Creagan et al, 1990; Barth et al, 1995; Keilholz et al, 1998), or to develop hypotheses, but these analyses were performed retrospectively, on data from patients treated within clinical trials performed with another justification.

Regarding adjuvant treatments, an example on how misleading survival data from uncontrolled phase II trials can be is the situation in the field of high-dose chemotherapy for patients with breast cancer. Comparison of phase II survival information with historical controls had suggested a survival benefit achieved with high-dose chemotherapy, however, this was not reproducible in randomized studies, because in the randomized studies the survival data of conventional treatment were much better than the historical controls.

An example regarding the change of control groups over time even in controlled randomized studies is illustrated in the development of adjuvant high-dose interferon treatment for melanoma. When the ECOG analysed their trial 1690 (Kirkwood et al, 1999), which had been designed to confirm the trial 1684 (Kirkwood et al, 1996), the survival curves of the high-dose interferon arms of both trials were found to be identical. However, the survival curve of the control group of the 1690 trial was identical to the treatment group, and significantly better than the control group of the 1684 trial. Numerous factors are being discussed, which may have caused the improved survival of the control group in the newer study. As this discussion is ongoing, it is evident that comparison with historical controls is not appropriate for drawing conclusions from adjuvant treatment studies.

A third important example is derived from the development of combination chemotherapy for patients with metastatic melanoma. It took 15 years from the initial favourable report of the four-drug regimen DBCT (dacarbacine, BCNU, cisplatin, tamoxifen, delPrete et al, 1984) to perform the decisive randomized study proving that this combination did not result in improved survival in patients with advanced melanoma (Chapman et al, 1999). However, during these 15 years the DBCT regimen had been widely adopted on the basis of uncontrolled phase II data.

### CONCLUSIONS

There is no evidence that patients with melanoma may benefit from adjuvant chemotherapy and the report by McClay et al does not change this situation. This uncontrolled study only shows the overall outcome, in terms of disease-free survival and survival, with surgically resected melanoma of stages II, III and IV. The patients represent a selected patient population, as they all had an initial performance status of 0 and an unspecified interval between surgery and initiation of chemotherapy. Such descriptive survival curves do not allow for any firm conclusions to be made regarding efficacy of adjuvant therapy. In order to develop efficient treatments for melanoma patients, we should all continue to make every possible effort to enter patients into controlled adjuvant trials.

### REFERENCES

Agarwala SS, Ferri W, Gooding W and Kirkwood JM (1999) A phase III randomized trial of dacarbazine and carboplatin with and without tamoxifen in the treatment of patients with metastatic melanoma. *Cancer* **85**(9): 1979–1984

Ahman D, Creagan E, Hahn R, Edmonson J, Bisel H and Schaid D (1989) Complete responses and long-term survivals after systemic chemotherapy for patients with advanced malignant melanoma. *Cancer* **3**: 224–227

Balch C (1992) Cutaneous melanoma: prognosis and treatment results worldwide. *Semin Surg Oncol* **8**: 400–414

Cocconi G, Bella M, Calabresi F, et al (1992) Treatment of metastatic malignant melanoma with dacarbazine plus tamoxifen. *New Engl J Med* **327**: 516–523

Creagan E, Schaid DDLA and Frytak S (1990) Disseminated malignant melanoma and recombinant interferon: Analysis of seven consecutive phase II investigations. *J Invest Dermatol* **95**: 188S–192S

Creagan ET, Dalton RJ, Ahmann DL, et al (1995) Randomized surgical adjuvant clinical trial on recombinant interferon-alfa-2a in selected patients with malignant melanoma. *J Clin Oncol* **13**: 2776–2783

DelPrete SA, Maurer LH, O'Donnel J, Forcier FJ and Le Marbre P (1984) Combination chemotherapy with cisplatin, carmustine, dacarbazine and tamoxifen in metastatic melanoma. *Cancer Treat Rep* **68**: 1403–1405

Jungnelius U, Ringborg U, Aamdal S, Mattsson J, Stierner U, Ingvar C, et al (1998) Dacarbazine-vindesine versus dacarbazine-vindesine-cisplatin in disseminated malignant melanoma. A randomised phase III trial. *Eur J Cancer* **34**(9): 1368–1374

Keilholz U, Conradt C, Legha S, Khayat D, Scheibenbogen C, Thatcher N, et al (1998) Results of IL-2-based treatment in advanced melanoma: a case-record based analysis of 631 patients. *J Clin Oncol* **16**(9): 2921–2929

Keilholz U, Goey S, Punt C, Proebstle T, Salzmann R, Scheibenbogen C, et al (1997) IFNa/IL-2 with or without Cisplatinum in metastatic melanoma: a randomized trial of the EORTC Melanoma Cooperative Group. *J Clin Oncol* **15**(7): 2579–2588

Kirkwood JM, Strawderman MH, Ernstoff MS, Smith TJ, Borden EC and Blum RH (1996) Interferon alfa-2b adjuvant therapy of high-risk resected cutaneous melanoma: the Eastern Cooperative Oncology Group Trial EST 1684. *J Clin Oncol* **14**: 7–17

Kirkwood JM, Ibrahim J, Sondak V, et al (1999) Preliminary analysis of the E1690/S9111/C9190 Intergroup Postoperative Adjuvant Trial of high- and low-dose IFNalpha2b (HDI and LDI) in high-risk primary or lymph node metastatic melanoma. *Proc Am Soc Clin Oncol* **18**: 2072(abstr)

McClay EF, Mastrangelo MJ, Bellet RE and Berd D (1987) Combination chemo/hormonal therapy in the treatment of malignant melanoma. *Cancer Treat Rep* **71**: 465–469

McClay EF, Albright KA, Jones JA, Christen R and Howell SB (1993) Tamoxifen modulation of cisplatin sensitivity in human malignant melanoma cells. *Cancer Res* **53**: 1571–1576

McClay EF, McClay MET, Monroe L, Baron PL, Cole DJ, O'Brien PH, Metcalf JS and Maize JC (2000) The effect of tamoxifen and cisplatin on the disease free and overall survival of patients with high-risk melanoma. *Br J Cancer …* **xx**: 00–00

Rusthoven J, Quirt I, Iscoe N, et al (1996) Randomized, double-blind, placebo-controlled trial comparing the response rates of carmustine, dacarbazine, and cisplatin with and without tamoxifen in patients with metastatic melanoma. *J Clin Oncol* **14**: 2083–2090

Saxman S, Meyers M, Chapman P, Destro A, Panageas K, Begg C, et al (1999) A phase III multicenter randomized trial of DTIC, cisplatin, BCNU ad tamoxifen versus DTIC alone in patients with metastatic melanoma [Abstract]. In: Proceedings of ASCO, 1999. *Melanoma and Sarcoma* 536a

© 2000 Cancer Research Campaign

